# Behaviour Change Domains Likely to Influence Occupational Therapist Use of the Canadian Occupational Performance Measure

**DOI:** 10.1155/2020/3549835

**Published:** 2020-05-08

**Authors:** Heather L. Colquhoun, Rafat Islam, Katrina J. Sullivan, Jane Sandercock, Sandy Steinwender, Jeremy M. Grimshaw

**Affiliations:** ^1^Department of Occupational Science and Occupational Therapy, University of Toronto, 160-500 University Ave, Toronto, Ontario, M5G 1V7, Canada; ^2^Ottawa Hospital Research Institute, Clinical Epidemiology Program, The Ottawa Hospital, General Campus, 501 Smyth Road, Centre for Practice Changing Research, Ottawa, Ontario, K1H 8L6, Canada; ^3^University of Western Ontario, PhD Candidate Health Information Science, Health Sciences, London, Ontario, N6A 5B9, Canada; ^4^Department of Medicine, University of Ottawa, 451 Smyth Road, Ottawa, Ontario, K1H 8M5, Canada

## Abstract

**Introduction:**

Occupational therapists have shown low adoption rates for many evidence-based practices. One such practice is the limited uptake of standardized outcome measures such as the Canadian Occupational Performance Measure. Use of this measure has not consistently translated into practice despite decades of encouragement. Theory-based approaches to understanding healthcare provider behaviour change are needed if we are to realize the goal of attaining practice that is in keeping with evidence. This study utilized the Theoretical Domains Framework, a theory-based approach for understanding barriers to evidence-based practice, in order to increase our understanding of the limited uptake of the Canadian Occupational Performance Measure in occupational therapy practice.

**Methods:**

Theoretical Domains Framework methods were followed. First, primary data was collected from occupational therapists through semistructured interviews that focused on key behaviour change domains as they related to the use of the Canadian Occupational Performance Measure. Two independent researchers coded interview data into domains, derived belief statements from the data, and used belief strength, conflict, and frequency to determine the more and less influential domains for using the Canadian Occupational Performance Measure.

**Results:**

Interviews with 15 practicing occupational therapists across a range of practice areas yielded six key behaviour change domains for increasing the use of the Canadian Occupational Performance Measure. The more relevant domains were *Social influences*, *Social professional role and identity*, *Beliefs about consequences*, *Beliefs about capabilities*, *Skills*, and *Behavioural regulation*). The other eight domains were found to be less relevant.

**Conclusion:**

We identified important domains and beliefs that influence the use of the Canadian Occupational Performance Measure by occupational therapists. Results inform our understanding of the use of this measure in practice and identify potential targets for behaviour change interventions.

## 1. Introduction

The routine use of outcome measures by occupational therapists is viewed as an essential and evidence-based component of practice [1]. Using standardized measures can promote treatment planning [2], document the impact and efficiency of services [3], and promote meaningful communication of patient progress [2]. Using outcome measures has generally been valued by clinicians [3–5] and has been encouraged and promoted for decades [1, 6], and yet, the use of outcome measures as a routine aspect of occupational therapy practice remains limited [7–10].

Accurate expectations for outcome measure use rates in occupational therapy remain challenging as research in this area has been limited, and use rates are typically based on a self-report survey [9–11]. Of these self-report surveys, studies indicate that use ranges from as low as 1% [12] to as high as 10% [11]. Studies on use rates presenting hypothetical client cases have found higher rates of up to 44% [10]. Actual use rates, as evidenced by chart review or observation, to our knowledge have not been published.

Similar trends of use are found for the Canadian Occupational Performance Measure (COPM) [13]. The COPM is a semistandardized and gold standard occupational therapy instrument designed to determine relevant occupational performance issues and measure client perceptions of change in performance and satisfaction with those issues over time [13]. Despite its relevance to occupational therapy, and approximately 30 years of encouragement to therapists to adopt the measure including knowledge translation efforts to support COPM use such as e-learning modules (http://www.thecopm.ca/learning-module/), use rates specific to the COPM are surprisingly low [12]. When prompted in a survey for desired assessment of participation post stroke, 2-4% of occupational therapists suggested the COPM [14]. In the delivery of occupational therapy for cerebral palsy, a higher rate of 26% intended use of the COPM was found, although this was based on self-report [10]. Evidence in support of verified COPM use rates in routine clinical practice is limited.

A systematic review to examine the barriers and facilitators to outcome measure use by allied health professionals (i.e., occupational therapy, physiotherapy, and speech language pathology) found 15 studies [3]. Key barriers included knowledge and confidence with using outcome measures, time limitations, and the degree of peer and organizational support for using measures. To date, studies indicate that alleviating these barriers does not result in sustained and routine use of standardized outcome measures [4, 15]. In addition, there remain discrepant views and attitudes towards outcome measurement by therapists. Colquhoun (2010) interviewed occupational therapists after they had been required to administer the COPM for 5 months. The therapists perceived and indeed experienced many benefits from using the COPM (e.g., care more focused on occupation and more client centredness) and recommended the routine use of the COPM. Yet, overall, the occupational therapists did not plan to continue using the COPM after study completion. Other surveys have found the same favourable attitudes to outcome measure use but with a lack of reported use rates in the same sample [5]. In a survey study examining occupational therapist attitudes and measurement use rates, the respondents cited lack of time as a barrier to standardized outcome measure use; yet, at the same time, 90% of the respondents reported the use of nonstandardized measures [11]. Efforts to better understand these barriers are needed to guide the development of interventions designed to improve adoption rates.

Our understanding of why the COPM has seen such limited uptake in occupational therapy practice remains limited, particularly with respect to beliefs and attitudes that could be amenable to change. One approach to barrier identification that targets these issues is the Theoretical Domains Framework (TDF) [16, 17]. The TDF is a broad-based theoretical framework that condenses 128 constructs from 33 key behaviour change theories into a set of 14 domains of behaviour change. The framework assists in the identification of which domains, and the associated modifiable beliefs, appear most crucial for changing a behaviour. The objective of this study was to use the TDF to improve our understanding of what beliefs influence the use of the COPM by occupational therapists.

## 2. Methods

We conducted a TDF study [17]. The overall approach includes mapping interview utterances to the TDF domains, developing belief statements consistent with utterances, and analyzing belief statements for their relevancy to changing the target behaviour (i.e., increasing the use of the COPM by occupational therapists). Ethics approval was obtained from the Ottawa Health Science Network Research Ethics Board, Ottawa, Canada (#20130367-01H).

### 2.1. Participants

Using purposive sampling, we recruited occupational therapists across all services in the occupational therapy department of a large urban health network in Ontario, Canada, that included several sites and therapists in various departments practicing in acute care, inpatient, and outpatient occupational therapy. All therapists were eligible who carried out any amount of clinical caseload and worked in any clinical area. Consistent with TDF studies, we aimed to interview approximately 12 therapists whose practice context would support using the COPM.

### 2.2. Data Collection and Procedures

The TDF interview guide was developed and pilot tested to elicit an understanding of participant beliefs related to the 14 domains in the 2012 version of the TDF [17]. The 14 domains are as follows: *Knowledge*, *Skills*, *Social professional role and identity*, *Beliefs about capabilities*, *Optimism*, *Beliefs about consequences*, *Reinforcement*, *Intentions*, *Goals*, *Memory/Attention decision processes*, *Environmental context and resources*, *Social influences*, *Emotion*, and *Behavioural regulation* (see Table 1 for a summary of domain definitions). In order to design the guide, we drew upon the expertise of our team in conducting TDF interviews (HLC, RI, and JMG) as well as publications of TDF studies that included interview guides that targeted health care provider behaviour change [18]. Example questions included the following: to understand *Beliefs about consequences*, we asked, “What do you think will happen if you use the COPM with your inpatients, both positive and negative?”; and to understand *Social influences*, we asked, “Would any other team members influence whether or not you use the COPM with your inpatients?” and “How does the patient affect your decision to use the COPM?” We did not ask participants if they used the COPM as we were aware that the COPM was being used minimally in the study environment and we believed that if we directed their attention to low compliance this could increase self-presentation bias and lead to participants erroneously focusing on external factors [19] (see Supplemental file 1 for a copy of the interview guide).

Potential participants received information regarding the study during a staff meeting and were asked to contact the research team if they were interested in being interviewed. All participants provided informed consent. The interviews were face-to-face and lasted approximately 30-45 minutes.

### 2.3. Data Analysis

Our approach to analysis including the framework for the coding process followed several published TDF studies that were part of a special TDF thematic series in *Implementation Science* and attempted to understand healthcare delivery practices from the viewpoint of healthcare providers [18, 20]. The first step was to transcribe the interviews. Next, using NVIVO, two researchers independently coded utterances from the interviews into TDF domains (e.g., Social influences and Beliefs about capabilities). The two first transcripts were used to establish an understanding between the two coders as to how the utterances in the interviews should be coded into the 14 TDF domains. This coding occurred in tandem. Discrepancies were discussed until both coders were satisfied that a mutual understanding existed for the coding. Coding occurred independently for all transcripts from that point on. The final coding was completed by comparing all codes and using discussion to come to consensus on differences. All coding was done independently by two of HLC, RI, SS, and JS.

The next step involved transferring the coded utterances, organized by domain, from NVIVO into Excel and developing a corresponding belief statement for each utterance coded into a domain. The belief statements provide detail about the perceived role of the domain in influencing the behaviour. If multiple utterances result in similar belief statements, patterns of consistencies and differences will emerge related to the belief statements. As an example, utterances such as “Using the COPM takes practice,” “I think using the COPM is more about practice than experience,” and “The more you use the COPM, the more information you get from it” could all be grouped under the belief statement “Using the COPM takes practice.” As another example, utterances such as “Normally I meet the patient and then decide if it's an appropriate tool” and “Using the COPM will depend on the nature of the client” could be grouped under the belief statement “My use of the COPM is influenced by my client.” Determining the belief statements was achieved by two coders independently developing the statements, followed by consensus discussions to agree on final belief statements that both coders agreed were indicative of the utterance. Once consensus on all belief statements was achieved, belief statements in each domain were grouped into similar beliefs. The final list of the most relevant domains and beliefs (i.e., the factors most likely to influence the behaviour of using the COPM) was decided based on consensus of two coders and was conducted in keeping with recommended TDF analyses. This included having coders iteratively discuss the groupings of belief statements as well as the following three criteria: (1) frequency of the beliefs across interviews (i.e., how many belief statements per domain), (2) presence of conflicting beliefs (i.e., different belief statements within a domain that indicated opposing beliefs), and (3) perceived strength of the beliefs impacting the behaviour (i.e., coder perceptions on how strong the specific beliefs appeared to be including the language used by participants such as “important” or “never” or “always”). Note that while each of the three criteria is relevant, each is considered in the context of the other two (e.g., while frequency is important, it does automatically indicate most relevant).

## 3. Results

Fifteen occupational therapists were recruited across the participating health network. Therapists worked predominantly in inpatient capacities (13/15 or 87%) with the remaining two working with outpatients. Of the therapists working with inpatients, four of these worked in inpatient psychiatry (4/13 or 31%) and nine (9/13 or 69%) provided longer term rehabilitation. Participants were all female and predominantly experienced therapists, having worked an average of 19.2 years (minimum of 10 years and maximum of 27 years) as an occupational therapist. About a half of the therapists had been working more than 21 years as occupational therapists (8/15 or 53%).

Our study identified six domains that were most relevant to the use of the COPM (*Social influences*, *Social professional role and identity*, *Beliefs about consequences*, *Beliefs about capabilities*, *Skills*, and *Behavioural regulation*). The remaining eight domains were deemed as less relevant (*Knowledge*, *Environmental context and resources*, *Intention*, *Goals*, *Emotion*, *Reinforcement*, *Optimism*, and *Memory/Attention decision processes*) (see Table 2 for a summary of all domains and number of beliefs). In total, 828 beliefs were generated (see Table 3 for sample belief statements and sample quotes for the relevant domains). Below is a summary of the domains as well as the associated key beliefs with an emphasis on the more relevant domains.

### 3.1. Most Relevant TDF Domains (*N* = 6) and Key Beliefs

*Social influences* had the highest number of beliefs (*n* = 134), and the perceived strength of the effect of the beliefs on the behaviour was high. *Social influences* appeared to play a large role in determining whether the occupational therapists we interviewed chose to use the COPM. While the setting (e.g., level of acuity of patient and length of stay) and team dynamics (e.g., interest or support of other team members, specifically physicians) were mentioned as influences to using the COPM, the strongest social influence of whether the COPM was used appeared to be from the clients and not from other team members. Therapists seemed to make a decision to use or not use the COPM based on client characteristics or perceptions of how clients would respond to the measure. Therapists indicated various client characteristics that positively influenced COPM use such as using the COPM with clients who they knew have ongoing issues they wanted to improve and clients who were higher functioning and the value of the COPM for clients who were struggling with setting goals. Therapists also spoke about choosing to not use the COPM with clients whom they thought would have a negative reaction to the measure, whom they believed would become angry or agitated by it, or whom they felt were too low functioning or had inadequate insight to engage in the process. Therapists reported that the COPM has the potential to negatively impact rapport and limit therapeutic relationships, limiting their use of the COPM.

*Social professional role and identity* had a high frequency of beliefs (*n* = 101) and strength in the perceived role of the beliefs on the behaviour. Beliefs in this theme highlighted the individual nature of an occupational therapist approach to assessment. Specifically, these assessment practices are viewed as autonomous and individually determined. Therapists frequently expressed the desire to “get information” about a client in different ways that suited their own approach and philosophy, their setting demands, or the needs of the client. Many indicated that if they chose to use the COPM at all, it was in a modified way, based on their own individual need for information. Therapists appeared largely unaware of what their occupational therapy colleagues were doing in terms of assessment practices or outcome measure use. More than that, however, they described the issue of assessment in practice as fundamentally individual and that they would be reluctant to ever suggest how another therapist should conduct their assessment or use outcome measures. In the case of the COPM, there did not seem to be a sense that the profession would make a “group” decision to use the COPM or that they would choose to influence their colleagues to use the COPM. This is consistent with what was found in *Social influences*: the influences seemed to come from clients more so than other occupational therapists.

*Beliefs about consequences* had a high number of beliefs (*n* = 112), strength of beliefs in influencing the behaviour, and conflicting beliefs. Therapists indicated many positive consequences of using the COPM including the facilitation of goal setting, improved ability to get to “know” the client, an aid to introducing the occupational therapy role, and having a standard approach to measurement. However, they also indicated that the knowledge gained from the COPM did not give them the information that they needed as part of their practice setting demands and that no negative consequences would come from not using the COPM. They also anticipated that a consequence of using the COPM was negative client reaction; therapists believed that using the COPM with a certain type of client could negatively affect the therapeutic relationship. Again, this was consistent with what was found under *Social influences*: the perspective of the client was the most important consideration in deciding whether or not to use the COPM.

*Beliefs about capabilities* had a comparatively less frequent number of beliefs (*n* = 37) than other domains but enough strong and conflicting beliefs in influencing the behaviour to warrant inclusion as a relevant domain. The conflict is related to whether the COPM was hard or easy to use and their confidence and comfort in doing so. Therapists professed to know how to use the COPM and that using the COPM was easy and straightforward. Most said they were comfortable, confident, and capable using the measure and that it was easy to use with the “right” clients. However, they also described many instances in which they were not confident or comfortable in their abilities to use the COPM. They indicated that it could be difficult to use, as it required a great deal of practice and experience, and that they were not confident that they are using the COPM properly. They outlined numerous contexts in which they found that the COPM was difficult to administer such as clients who had cognitive impairment or who were challenged to set goals. *Skills* had a relatively lower frequency of beliefs (*n* = 39) as well but was included as one of the relevant domains as it had conflict in beliefs and beliefs that were consistent with our learnings from *Beliefs about capabilities*. Therapists indicated that the COPM required very few skills, was easy to use, and that anyone (even students) could use it. However, they also highlighted many skills that were necessary for using the COPM (e.g., interviewing skills, the ability to guide clients and keep them “on track,” and explaining the meaning of occupation).

*Behavioural regulation* had a comparatively lower number of beliefs (*n* = 32) than other domains but contained strong beliefs related to what might encourage the use of the COPM in practice. Therapists indicated that using the COPM requires planning and preparation but that if it was an established part of the assessment process and automatically embedded into their assessment protocols or structures (e.g., including the COPM on the electronic health record), COPM use would be facilitated.

### 3.2. Less Relevant Domains (*N* = 8)

*Environmental context and resource* contained a high number of beliefs (*n* = 96) but appeared to have limited influence on the behaviour (i.e., few strong beliefs and conflict in beliefs). There were certainly beliefs stated related to there not being enough time to do the COPM in stressful practice environments, but they appeared to have less influence on the behaviour than other domains.

*Intentions* and *Goals* each had a similar pattern: low number of beliefs, low strength of beliefs in influencing the behaviour, but the presence of conflicting beliefs. Indeed, these two domains showed generally only two types of beliefs: using the COPM was a high priority for some and low for others (*Goals*) and that some intended to use the COPM and some did not (*Intentions*).

While the domain of *Knowledge* had a high frequency of beliefs (*n* = 116), the beliefs did not appear to represent conflicting beliefs or beliefs likely to influence behaviour. Participants were able to describe the COPM and reasons why one might choose to use the COPM (e.g., maintaining a client-centred practice, assisting with goal-setting, and staying focused on an occupation-based scope of practice). Some participants believed that the COPM was reliable and valid, some did not, and most did not have a clear understanding of why using outcome measures was important.

The remaining domains (i.e., *Optimism*, *Reinforcement*, *Memory/attention and decision processes*, and *Emotion*) did not appear relevant in our sample.

#### 3.2.1. Discussion

Using semistructured interviews that probed 14 domains of behaviour change (i.e., the TDF), we interviewed 15 practicing occupational therapists and found six relevant domains for influencing the use of the COPM. These domains emerged as a result of how often a particular belief within the domain was endorsed, whether there were conflicting beliefs, and the perceived strength of the beliefs for influencing the behaviour. The relevant domains were *Social influences*, *Social professional role and identity*, *Beliefs about consequences*, *Beliefs about capabilities*, *Skills*, and *Behavioural regulation*. To our knowledge, this is the first TDF study specific to occupational therapy practice. The TDF appeared highly useful in this context and resulted in information that contributed to our understanding of the influences of COPM use. We recommend additional TDF studies as appropriate for other areas of practice that require implementation efforts.

While studies have examined the clinical applications of the COPM [21, 22], the effect of using the COPM on occupational therapy practice [2], the effect of using the COPM on client outcomes, and barriers and facilitators to using the COPM [21, 23], no studies to our knowledge have focused specifically on our understanding of the factors influencing the use of the COPM from a behaviour change perspective. Contrary to studies that use cross-sectional survey designs to establish barriers to outcome measure use [3], our study has uncovered a set of barriers that are less prone to self-report biases, theoretically supported, and specific to one outcome measure.

Our sample was predominantly experienced therapists. We do not know how or if results might have been different with a more varied or less experienced sample although a recently published analysis investigating factors that determined the use of the COPM indicated that years of experience was not among the relevant factors [24]. That said, the sample being relatively experienced is noteworthy. Future consideration should be taken to examine the role of experience in, for example, perceptions of the benefit of the COPM and how they might be shaped over years of practice.

Client attitudes, or more specifically therapists' perceptions of client attitudes, appear to play an influencing role in occupational therapists' decisions to use the COPM. This is not surprising, and indeed is quite positive, in a field that prides itself on being client-centred [25, 26]. However, it is not clear that these perceptions of our clients' attitudes are necessarily accurate. While we have often studied the COPM use from the perspective of the occupational therapist [4], scant work has been done to understand the use of the COPM from the client's perspective. In fact, some research specific to the COPM suggests that therapists are poor at truly understanding the perspective of their clients [27]. A better understanding of the perspective of clients that have used the COPM would be helpful.

Efforts to design interventions to increase the use of outcome measures including the COPM remain underdeveloped. A systematic review of interventions to increase the use of standardized outcome measures in allied health professionals found 11 studies; only three of which included occupational therapists, and none focused on the COPM [28]. TDF studies are meant to foster improved intervention design by functioning in combination with other tools that can suggest interventions for behaviour change that align with TDF domains [29, 30]. This approach to intervention design results in the building blocks of an intervention that is theory-based and has a clearly articulated causal pathway between the intervention components and the domains of behaviour change. This can decrease the likelihood that we apply interventions that do not target the important beliefs or domains. A common example of this is when knowledge-based interventions (e.g., educational workshops) are used when knowledge is not a barrier [31].

Using these processes to choose behaviour change strategies [29], our results point to strategies to increase COPM use. Our study found that occupational therapists have an individualistic approach to assessment and that being influenced by other occupational therapists to use the COPM is unlikely to lead to increased COPM use. Indeed, the main influencer seems to be the client. Operationalizing this social influence into an intervention designed to change practice would be challenging. The intervention would need to be some form of a patient-mediated strategy [32] that involves clients being aware of the COPM and its benefits, and then, clients would need to encourage its use. Achieving this would likely require a restructuring of the social environment to foster such client influence [29] but could be promising for increasing the use of the COPM.

Therapists' responses illustrated a level of conflict around whether they felt comfortable and skilled enough to use the COPM. The results of this study suggest that COPM messaging should emphasize that with the right training, skill development, and practice, the COPM can be used successfully. Therapists attempting to incorporate the COPM into practice likely need additional and ongoing education support. Our study suggests that running a brief workshop on the COPM to teach about the measure will not be effective unless the workshop is devoted to detailed and ongoing skill development. Occupational therapists need rehearsal, practice, and graded skill building [29], including how to manage the situation when you are not sure if your client will want to use the COPM. The domain *Behavioural regulation* is typically viewed as an important postintentional aspect of behaviour change (i.e., for therapists who already have intentions to use the COPM but need support and encouragement to engage in the behaviour [33]). For therapists that intend to use the COPM, automatic processes in place to support their behaviour change might be useful [29] (e.g., incorporating the COPM into the electronic health record, making the COPM part of the standard assessment protocol, and keeping COPM forms in convenient locations). Of note are knowledge translation strategies already in place to facilitate electronic documentation of the COPM (e.g., the COPM Web-App; http://www.thecopm.ca/).

A recent retrospective chart review conducted in Japan found a 37% rate of actual COPM use in a subacute rehabilitation context [24]. This impressive rate of “real-world” COPM use should lead us to feel optimistic about increasing COPM use rates. This study also found that two factors indicated greater likelihood of COPM use: higher cognitive status in patients and current use of the COPM. Thus, the probability of using the COPM increases with clients with higher levels of cognition and for those already using the COPM, implying that attitudes and intentions towards COPM use are critical. Our study did not find the *Intentions* domain to be highly relevant, but this could be due to the sample having lower levels of intention to use the COPM generally and therefore not enough strength or conflict in beliefs to focus our attention on this domain. Further TDF studies in different contexts, with less experienced therapists, and with verified COPM use rates would be of value. In addition, studies with a greater focus on intentions to use the COPM would improve our understanding of its role in COPM use.

Several limitations of this study should be noted. Using the TDF requires specification of the behaviour. In this case, it was occupational therapists using the COPM as a routine aspect of practice. While this has given us detailed information specific to the COPM, we are unable to transfer this barrier analysis to other measures within occupational therapy practice. Our sample was a highly experienced one; it might not be representative of all practicing therapists. However, it is uncertain as to how this might affect results; we might expect a more experienced sample to be more familiar with the COPM, more efficient in practice, and thus more adept at administering the COPM. Our sample was Canadian only, and although there is limited evidence to suggest that outcome measure use practices vary by country, it is possible that they do, and this could limit the international transferability. Our sample was from one (albeit large) occupational therapy department and thus reflects a limited perspective that could be influenced by a specific department culture. We chose to include all of the therapists in the department, regardless of the work area, but this may have resulted in greater breadth than depth. In addition, while we attempted to recruit as many therapists as we could, it is possible we did not achieve full saturation. We collected therapist information such as the area of service provided and number of years in practice, but we could have contextualized our results even more had we collected information such as therapist practice traits or courses taken to support use of measures. This study identified potential barriers that might influence this behaviour. We cannot establish the factors as actual barriers until we test them. Of note is that this TDF study was conducted prior to the publication of a TDF guide [34]. To that end, we point readers towards the TDF guide for future TDF studies. In addition, TDF domain definitions have been recently revised to improve clarity [33].

## 4. Conclusions

We utilized the TDF to identify important domains and beliefs that influence the use of the COPM by occupational therapists. Results inform our understanding of the use of this measure in occupational therapy practice and identify potential targets for behaviour change interventions aimed at increasing the use of the COPM by occupational therapists.

## Figures and Tables

**Table 1:** TDF domains and their definitions [17].

TDF domain	Definition
Knowledge	An awareness of the existence of something
Skills	An ability or proficiency acquired through practice
Social professional role and identity	A coherent set of behaviours and displayed personal qualities of an individual in a social or work setting
Beliefs about capabilities	Acceptance of the truth, reality, or validity about an ability, talent, or facility that a person can put to constructive use
Optimism	The confidence that things will happen for the best or that desired goals will be attained
Beliefs about consequences	Acceptance of the truth, reality, or validity about outcomes of a behaviour in a given situation
Reinforcement	Increasing the probability of a response by arranging a dependent relationship, or contingency, between the response and a given stimulus
Intentions	A conscious decision to perform a behaviour or a resolve to act in a certain way
Goals	Mental representations of outcomes or end states that an individual wants to achieve
Memory/attention decision processes	The ability to retain information, focus selectively on aspects of the environment, and choose between two or more alternatives
Environmental context and resources	Any circumstance of a person's situation or environment that discourages or encourages the development of skills and abilities, independence, social competence, and adaptive behaviour
Social influences	The interpersonal processes that can cause people to change their thoughts, feelings, or behaviours
Emotion	A complex reaction pattern, involving experiential, behavioural, and physiological elements, by which the individual attempts to deal with a personally significant matter or event
Behavioural regulation	Anything aimed at managing or changing objectively observed or measured actions

Note. TDF: Theoretical Domains Framework.

**Table 2:** Total number of beliefs per domain.

Domain	Total number of beliefs
*Social influence*	*134*
Knowledge	116
*Beliefs about consequences*	*112*
*Social professional role and identity*	*101*
Environmental context and resources	96
Memory, attention, decision processes	41
*Skills*	*39*
*Beliefs about capabilities*	*37*
Behavioural regulation	32
Intentions	30
Goals	28
Reinforcement	27
Emotion	24
Optimism	11
Total	828

Note. Italics indicate a relevant domain.

**Table 3:** Summary of relevant TDF domains with sample belief statements and sample quotes.

Relevant TDF domains	Belief statements	Sample quote
Social influences	(i) My use of the COPM is influenced by my clients.	(i) “Normally I meet the patient and then decide if [the COPM] is an appropriate tool.” (D) (ii) “[Using the COPM] really depends on the patient and what their, you know, how they present.” (D)
	(ii) Client characteristics can positively and negatively influence my use of the COPM.	(i) “Well again [using the COPM] depends on their diagnosis. If they had a chronic disability and there was ongoing issues for them that they wanted to work towards improving… I think that the [COPM] would be an appropriate tool.” (O) (ii) “If I'm having a hard time establishing goals with somebody then I would go to [the COPM].” (D) (iii) “Well they need to have insight, they need to be able to answer the questions independently…. and well they have to be cognitive.” (P) (iv) “Well the client [affects my decision to use the COPM]. I mean if the client is really profoundly impaired and I know that they have severe comprehension difficulties you know they may not be a person that I want to use [the COPM] on.” (A)
	(iii) Perceptions that the client will have difficulty with the COPM will negatively influence my use.	(i) “Well [I might use the COPM] except for that client that is very abrupt and… it would take me too much energy to get his anger down because he gets angry easily.” (C) (ii) “If [the client] starts to get the feeling [when using the COPM] that the outcome measure is more important [to me] and not what they are saying and they are reluctant to rate [the COPM] or they do not get it…I can lose them.” (I) (iii) “[The clients] can be depressed or… angry, they are in this spectrum so do you want to agitate the anger more [using the COPM]…oh yeah you cannot do all those things so then you are like precipitating more depression, right.” (J) (iv) “There are patients that when you say well I'm gonna ask you to rate on a scale of 1-10 how important it is or how you feel about it…they get angry because they say well what do you think, what a stupid question.. I cannot move anything on my right side and I can barely talk and then the patient would get angry so that did that stop me [using the COPM].” (Q)
	(iv) Other team members do not influence my use of the COPM.	(i) “Other team members do not influence my use of the COPM.” (A, B, E)

Social professional role and identity	(i) As an OT, I can decide what type of measurement tools to use.	(i) “I guess it's up to your discretion [using the COPM]…” (G) (ii) “I think there is room for choice and for you to decide what type of tools you want to use to measure a specific item.” (G)
	(ii) I can choose to modify the COPM as needed.	(i) “And then there's the [COPM] rating scale which sometimes I'll ask [the clients] to just leave.” (H) (ii) “…to be honest I rarely do it exactly the way it [the COPM] is supposed to be done.” (B)
	(iii) I am uncomfortable telling my peers what to use as an assessment.	(i) “So yeah I do not know how I would encourage the other team measures to use it. It's pretty hard I find to just assume somebody's gonna use it or I think everybody does their thing their own way so it's not something I'd push.” (E) (ii) “No, I do not know who uses [the COPM] in my department. It's not a discussion that I have with other people.” (A) (iii) “I did a study on the COPM, I have worked here for a long time. I have never presented, never been asked about its use and never have I done anything to assist the therapists who use the COPM even though my managers know of my experience and how I use it.” (I)

Beliefs about consequences	(i) The COPM offers benefits to clients.	(i) “I think it helps my clients to maybe understand what information is important to me. I think it gives the client an opportunity to actually talk about the things that they'd like to be able to do better or to be able to do; so it gets at their personal goals.” (A) (ii) “I'll use the COPM midway sometimes to ask them where they are at now and then I show what they were at the beginning and that encourages them.” (C) (iii) “I think there is the possibility that we could miss something and miss something that is important to them that maybe they do not have an opportunity or they do not feel like saying and then if you go through that [COPM] form maybe it would identify something.” (F) (iv) “I would say the costs [of the COPM] are worth it for the benefits that are achieved through a certain group of clientele.” (J)
	(ii) The COPM offers no benefits to clients.	(i) “If I was to look at my ABI clients over the last 4-5 years and pull out every COPM I've done the irony is I would find the same first 3 goals constantly, it is well 90% of the case.” (E) (ii) “There's lots of very, very skilled therapists that I work with who are excellent at their jobs and have never touched the COPM.” (A) (iii) “I'm not convinced that I would gain a lot more than what I'm actually doing right now [without the COPM].” (B) (iv) “I do not use it [the COPM] with my current clients and it's fine too.” (E) (v) “…you end up with some goals [when using the COPM], some of them are good, some of them you cannot really work on.” (F) (vi) “I do not feel that our patients are less, I do not think if I do not use it [the COPM] I do not think the clients are less what's the word getting less of a treatment session.” (F) (vii) “Nothing bad happens if somebody does not [use the COPM] because everybody is very skilled, very compassionate, very knowledgeable, motivated.” (Q) (viii) “I know it sounds bad but like who will be looking at the [COPM] like we have so many things to do like it's gonna not make a difference. I think if it was making a difference we would do the effort.” (P)
	(iii) The COPM takes too much time.	(i) “A negative aspect of using the COPM I think because of the time component with it then I probably would have to see less people.” (H)
	(iv) My clients will not like doing the COPM.	(i) “On the other hand, I can also get the opposite they get frustrated, angry, want to leave. I lose them in the interview which just means that I've pushed a bit hard and I'm pushing because I want the outcome. I want the numbers and they are not ready for it, cannot do it …and if they refuse after 3 questions then I've got to stop and then I may not get that opportunity again, right.” (I)

Beliefs about capabilities	(i) I am confident and comfortable using the COPM. (ii) The COPM is easy to use.	(i) “I'm fairly confident about it.” (A) (ii) “I find it quite straightforward it's easy to use.” (D) (iii) “I have used it with patients before so yes I am comfortable with it.” (J) (iv) “Using the COPM is easy.” (Q)
	(iii) I am not confident and comfortable using the COPM. (iv) The COPM is difficult to use.	(i) “It's not always easy to use.” (D) (ii) “Because it's so very seldom that I use it I would not say that it's easy to use.” (B) (iii) “If you want it to be standardized…to ask the question exactly how it's said…I was not able to do it.” (C) (iv) “It is more difficult because I even find that applying it is kind of like a process.” (F) (v) “So I think I have to educate myself a little bit more just improve my skills to use the COPM and feel comfortable with using the COPM.” (G) (vi) “…the hardest part about using the COPM is knowing the questions to ask. If you knew the questions to ask you could probably be more efficient using it.” (H) (vii) “I would have to practice a few times I have not used it in a long time.” (J)

Skills	(i) Few skills are needed to use the COPM.	(i) “But you know the fact that students are able to do it suggests to me that….you can use it [the COPM] without having a lot of prior experience.” (A) (ii) “Not a lot of experience is needed to use the COPM.” (P)
	(ii) Many skills are needed to use the COPM.	(i) “Well I think your interviewing skills are obviously important. Your ability to keep the client on track is important. You know sometimes when you are talking about their previous life, sometimes they start crying…and there's this element of being able to continue on with the [COPM] interview, not trying to avoid it because you know that the client's uncomfortable with it.” (A) (ii) “I think you need some clinical judgement and you know to be able to guide the patient on what might be realistic and not realistic depending on their overall condition.” (D) (iii) “I think you need to be fairly experienced in your interviewing…in order to really just use the COPM in its form that it's in now I think you'd probably need to have 2-3 years of experience.” (H) (iv) “…the patients do not get the concept of occupation. And so it's very hard as an OT… it's not [just] using the COPM it's going that one step further beyond to talk about occupation.” (I) (v) “[The skills needed are…] interview skills, listening skills, knowing how to interview skills, knowing how to ask the question and giving the individual time to answer it.” (Q) (vi) “I think you need some degree of clinical reasoning to be able to pull information you have gathered through the COPM and apply it to your interventions.” (R)

Behavioural regulation	(i) Automatic processes for documenting the COPM after completion on the chart would increase my use of the COPM.	(i) “[What helps is] the fact that there's a place on my initial assessment and then the discharge summary where I can sort of record the information fairly easily that I have information already that I just cut and paste you know a little blurb about the COPM.” (A) (ii) “…who knows it might be actually you know you might be able to complete it on an iPad or something like that.” (A) (iii) “I think it would have to be I would have to include it in my practice like in a structured way to that it's not an extra like thought for me or an extra step it's just it would be part of the assessment.” (R)
	(ii) Planning is necessary to use the COPM.	(i) “I think for me it [the COPM] just needs to be a little bit more in my face somehow like I need to have it be considered you know part of my plan.” (D) (ii) “It would take a whole lot of planning ahead.” (R)

Notes. TDF: Theoretical Domains Framework; COPM: Canadian Occupational Performance Measure; OT: occupational therapist.

## Data Availability

Our Research Ethics Board approval does not permit sharing of interview transcripts. Excel documents indicating beliefs statement are happily shared on request.

## Supplementary Materials

Supplementary Materials Supplemental file 1 is a copy of the interview guide.
