# Supplementary material for: Behaviour Change Domains Likely to Influence Occupational Therapist Use of the Canadian Occupational Performance Measure
Source: Occup Ther Int. 2020 May 8;2020:3549835. doi: 10.1155/2020/3549835 (PMC7245666; doi:10.1155/2020/3549835)
Supplement: Supplementary Materials — Supplemental file 1 is a copy of the interview guide. [file 3549835.f1.docx]

**Semi-Structured Interview Guide with Occupational Therapists for routinely using the Canadian Occupational Performance Measure for clients in inpatient rehabilitation environments**

**Introduction**: *The general aim of the interview is to help us understand* *how you make decisions to use the Canadian Occupational Performance Measure (COPM) as a routine part of your care with clients that receive inpatient rehabilitation from you. We want to know what influences your decision to use the COPM with your clients. There are no right or wrong answers; we are trying to understand how different therapists approach this issue, so please answer frankly.*

I’d like to start with some basic questions about your practice:

1. What type of setting do you practice in? (type of patients)
2. How many years have you worked as an Occupational Therapist?
3. Please describe the typical process you undertake for completing an initial assessment with your clients.
4. Describe for me the type of client you believe should have the COPM used with them?
5. On average, how many clients that you see per month patients would you identify as fitting each of these descriptions? (or percentage).

*Thank you. For the rest of the interview, I have some slightly more specific questions about what factors influence how you make decisions to use the COPM for your inpatients. Some questions may seem repetitive, but please bear with me as the questions are derived from a specific way of understanding human behaviour and I want to cover all important areas.*

Are you ready to get started?

Set the scene:

When you have clients come onto your caseload, you need to start with an assessment of their status in order to develop your treatment plan. Typically, standardized outcome measures, including the Canadian Occupational Performance Measure – or COPM - are recommended as part of this process. I’m asking you to think about the situation where you are seeing a client for an initial assessment of their status........

**Nature of the Behaviour**

1. **When you complete an initial assessment, tell me how you go about doing it.**

**Knowledge**

1. Are you aware of any guidelines (national, provincial or institutional) or recommendations about using the COPM with your inpatients?
2. What is your understanding of the guidelines or recommendation? **(Prompt –what do they say?)**
3. Do you believe these guidelines/recommendations to be evidence based? What is your interpretation of the evidence?
4. Do you think the COPM is a useful instrument or not? (Prompt: Expand on rationale).
5. What characteristics about the COPM do you find useful? (Prompt: Are there any key characteristics you think are useful?)
6. Is the COPM a valid and reliable instrument?
7. What is a valid and reliable instrument?
8. What is a clinically useful instrument?

**Skills**

1. How much expertise or experience do you think one needs in order to use the COPM in inpatient rehabilitation?
2. Are there any skills or techniques that would help you use the COPM in inpatient practice? What are they?

**Memory, Attention and Decision Processes**

1. Is using the COPM with your inpatients an automatic part of your job or is it something you take time to think about when you do an assessment? **(Prompt: routine, automatic. If you don’t use it at all, do you decide not to automatically or do you consider using it?)**
2. Is it typically an easy or difficult decision to make? **(Prompt: Weigh pros and cons etc.)**

**Social/Professional role & identity**

1. Is using the COPM with your inpatients a standard part of your assessment?
2. Is there anything in your professional role that influences whether you use the COPM with your inpatients? **(Prompt: professional training, a protocol)**

**Environmental Context & Resources**

1. What aspects of your clinical setting influence whether you use the COPM? (Prompt: Is there anything about your setting that influence you using the COPM or not using it?)
2. Are there any resource factors in your clinical setting that influence whether you use the COPM?
3. Are there any competing tasks or time constraints that might influence whether or not you *use the COPM?*

**Intention**

1. Do you try to use the COPM with all of your clients?

**Behavioural Regulation**

1. Do you expect you will *use the COPM with your inpatients?*
2. **If no to question 25…**

Why? What usually prevents you from following through with using the COPM?

**If yes to question 19…**

Why? What will help you follow through with using the COPM?

1. What are some personal ways/steps of working that would help you to use the COPM?
2. Do you make specific plans to use the COPM (for example, plan a time to use it, keep the assessment sheets handy)?
3. If you wanted to implement changes in your own practice (individual/team setting/practice setting) to encourage using the COPM, what do you think would be the steps necessary to do this?
4. What kind of instrument would you want to use?

**Optimism**

1. In general, what do you think will happen if you *use the COPM with your inpatients?* **(*prompt: to patients, to colleagues, yourself, short and long term) To what extent do you think those things actually happen?***
2. In general, what do you think will happen if you don’t use the COPM for your clients? **(*prompt: to patients, to colleagues, yourself, short and long term) To what extent do you think those things actually happen?***

**Goals**

1. Do you want to use the COPM with your inpatients?
2. Considering the other priorities you may have, how important is it to you to use the COPM with your inpatients? (Scale of 1-10)
3. Did you feel that you need to use the COPM with your inpatients? Why or why not?
4. What do you think you will learn/understand or ‘get’ from using the COPM? (Prompt: How important is that to you?)

**Reinforcement**

1. Are there any personal incentives that have worked for you in the past that help you use the COPM with your inpatients?
2. Are there any incentives given by others that have worked for you in the past that help you use the COPM with your inpatients?

**Beliefs about Capabilities**

1. How easy or difficult is it for you personally to use the COPM with your inpatients?
   1. If difficult, what issues have they encountered that make use the COPM with your inpatients problematic?
   2. If easy, what has helped make your using the COPM with your inpatients easy?
2. Are you confident that you are able to properly use the COPM with your inpatients?
3. Are you comfortable using the COPM with your inpatients with this patient population? Why or Why not?

**Social Influences**

1. Would any other team members influence whether or not you use the COPM with your inpatients? **(Prompt: who else; Other OT’s, other rehab clinicians, any other team members, managers, practice leads)**
2. How does the patient affect your decision to use the COPM? **Prompt: “If they appear particularly interested in their care or ask for outcome information - would that influence whether you use the COPM with them?”**
3. Do your colleagues generally agree with you on this issue?

**Emotion**

1. When you think about using the COPM with your clients, what feelings do you experience?
2. Do your emotions ever influence whether you use the COPM with your inpatients?

**Beliefs about Consequences**

1. What do you think will happen if you use the COPM with your inpatients, both positive and negative? **(prompt: Speak specifically in terms of to patients, to colleagues, to yourself, short and long term)**
2. What are the benefits to patients to use the COPM?
3. What are the drawbacks to patients to use the COPM?
4. Do you believe the costs of using the COPM are worth the benefits? Why or why not?

*That’s all the questions I have for you; has anything occurred to you about this topic that we haven’t asked about?*

*Are there any clarifications at this point you would like to make? Thank you!*
